# Application and automation of flow injection analysis (FIA) using fast responding enzyme glass electrodes to detect penicillin in fermentation broth and urea in human serum

**DOI:** 10.1155/S1463924692000269

**Published:** 1992

**Authors:** Helmut Meier, François Lantreibecq, Canh Tran-Minh

**Affiliations:** Laboratoire de Biotecknologie Ecole des Mines de Saint-Etienne Saint- Etienne Cedex 02 42023 France

## Abstract

An enzyme immobilization technique has been developed to determine the concentration of biological compounds. This technique has been applied to penicillinase and urease, which are crosslinked as very fine films directly onto the sensitive ends of pH glass electrodes, thereby dispensing with the need of an on-line enzyme reactor. The biosensor is incorporated in an FIA system within a magnetically stirred detection cell. Penicillin-V in fermentation broth and urea in human serum samples were detected and the results were compared with HPLC and spectrophotometric methods. On-line measurement is achieved through the automation of this FIA system.


Journal of Automatic Chemistry, Vol. 14, No. 4 (July–August 1992), pp. 137–143

### Application and automation of flow injection analysis (FIA) using fast responding enzyme glass electrodes to detect penicillin in fermentation broth and urea in human serum

**Helmut Meier, François Lantreibecq and Canh Tran-Minh**

*Laboratoire de Biotechnologie, Ecole des Mines de Saint-Etienne, 42023 Saint-Etienne Cedex 02, France*

*An enzyme immobilization technique has been developed to determine the concentration of biological compounds. This technique has been applied to penicillinase and urease, which are cross-linked as very fine films directly onto the sensitive ends of pH glass electrodes, thereby dispensing with the need of an on-line enzyme reactor. The biosensor is incorporated in an FIA system within a magnetically stirred detection cell. Penicillin-V in fermentation broth and urea in human serum samples were detected and the results were compared with HPLC and spectrophotometric methods. On-line measurement is achieved through the automation of this FIA system.*

### Introduction

Samples containing penicillin or urea are frequently measured in pharmaceutical and clinical laboratories. The use of a conventional combined pH glass electrode as the enzyme electrode would provide an attractive and versatile sensor because many enzymatic reactions involve hydrogen proton formation (enzyme reaction by penicillinase) or consumption (enzyme reaction by urease). It is possible to determine penicillin and urea by immobilizing penicillinase or urease onto glass electrodes. The principle of operation of the sensor is based on the changes in the $H^+$ concentration resulting from the enzymatic hydrolysis of penicillin by penicillinase (see figure 1(*a*)) and urea by urease (see figure 1(*b*)).

Since Flow-injection analysis (FIA) is characterized by its simplicity, economy, high sample throughput and great versatility, its combination with an enzyme sensor provides an excellent technique for solving problems in such areas as bioprocesses [1,2], clinical studies [3], food analysis [4], drug analysis [5] and environmental studies [6].

FIA has been successfully applied for the determination of numerous liquid-phase compounds. The technique can be considered as an analysis in the fixed-time mode. Thus, the coupling of biosensors with flow-injection analysis allows fast detection and reduces the cost per determination. Immobilized enzymes have found widespread application in FIA [7].

Reports of urea detection in body blood by enzymatic reaction using FIA have been published [3,8,9]. The

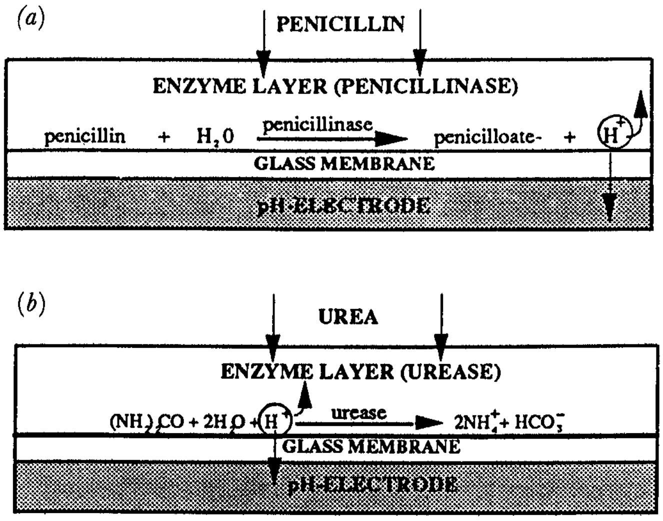

*Figure 1.(a) The penicillinase glass electrode; (b) The urease glass electrode.*

ammonia produced was detected by the pH changes arising from the hydrolysis of urea in immobilized urease reactor [8], or spectrophotometrically by ammonia conversion to an indophenolate dye [3]. A fluorescence method for the determination of urea has also been reported – urease was immobilized on 2-Fluoro-1-Methylpyridinium salt-activated fractogel [9].

Several types of enzyme electrodes for the determination of penicillin in batches have been developed and are described elsewhere [10]. Penicillin detection based on the enzymic hydrolysis by coupling an immobilized enzyme reactor with an electrochemical detector have been reported [11–13]. In all cases the electrochemical detector was a pH electrode.

A penicillin sensor has been incorporated in an FIA system, without using an enzyme reactor, which ensured a high sample throughput [2]. This detection system has possibilities for on-line monitoring of penicillin fermentation processes and also for the routine determination of urea in body fluids.

The present paper describes the automation and application of the FIA system in which a urea electrode and a penicillin electrode are directly incorporated in a homemade stirred-flow detection cell. The sample is diluted in

0142–0453/92 $3.00 © 1992 Taylor & Francis Ltd.

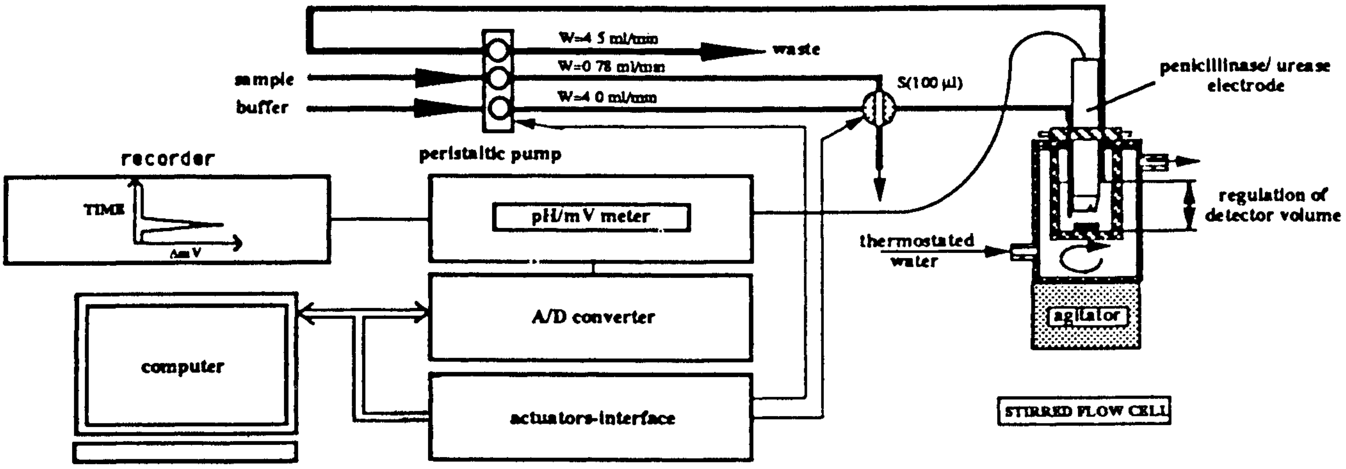

*Figure 2. Experimental set-up for the FIA system using fast responding enzyme glass electrodes to detect penicillin and urea.*

the cell by mixing with a buffer solution (figure 2). Urease and penicillinase were immobilized onto a combined pH glass electrode. This sytem was used to detect penicillin in fermentation samples manually and urea in human serum measured by the automated system. Various parameters influencing the electrode response were studied and the performances of penicillin sensor and the urea sensor were compared.

### Experimental

### *Hardware*

*Detection cell and agitator*

The configuration is shown in figure 2. Penicillinase and urease were immobilized over a combined pH-glass electrode: type LoT 403-M8-S7 (Ingold, Paris). A home-made stirred-flow cell is used as detection chamber. PVC and PTFE were used to construct the cell; small stainless-steel tubes were used as fluid inlets (in order to connect the tubes to the cell). The potentiometric measurements were obtained using a Radiometer pHM-64 research pH meter connected to a SEFRAM-Recorder (Sefram-Servotrace, Paris) and to the computer. The detection cell was agitated by a stirrer (MIKROLAB, Aarhus, Denmark) at a moderate speed.

*Pumps and tubing*

Sample and buffer solutions were pumped by two two-channel peristaltic pumps (ISMATEC SA, Zürich, Switzerland) using Tygon tubes 0·51, 1·42 and 1·52 mm inner diameter (Bioblock, Illkirch, France).

*Injection valve and actuator*

Automatic sample injection is carried out by use of a timing control pneumatic actuator connected to a four-way teflon injection valve (both Rheodyne, Cotati, USA). The actuator permits automatic operation of the valve.

*Automation elements*

The analogue minivolt signal arising from the pH-meter is converted to digital equivalent by a 6B11 module mounted on a 6BP04-2 backplane. The digital I/O backplane is a 6B50-1 module which provides 24 digital I/O channels – these backplanes are from Analog Devices (Norwood, USA). The I/O channels are connected to solid-state relays plugged in a EGS08000 backplane from Celduc (Sorbiers, France). These relays control the injection valve and the pumps.

The microcomputer (Macintosh Plus) is connected to the 6BPO4 backplane via the serial port (RS232).

### *Software*

The software is written in Quick Basic, and it is structured with subroutines for control of the injection valve and the pumps, data acquisition, calibration, and calculations. Subroutines ensure recording of the base-line, injection of sample, recording of response signal and preparation of the next sample injection. The record signals are saved, and, by means of calculation and calibration subroutine, the mV-peak height is converted to the appropriate concentration of the substrate. Four calibration samples with different concentrations are used to trace the calibration curve.

### *Reagents*

0·01 M sodium-phosphate buffer ($NaH_2PO_4$ and $Na_2$-$HPO_4.2H_2O$, pH 6.5 from Riedel-de Haën, Germany) and 0.005 M sodium-phosphate buffer ($NaH_2PO_4$ and $Na_2HPO_4.2H_2O$, pH 7·75, also from Riedel-de Haën, containing 0.1 M NaCl, were used as the working buffers. For the preparation of the urea and the urease solutions, the 0·005 M sodium-phosphate buffer was used. The Penicillin-V solutions were prepared in a fermentation broth composition [14], the penicillinase solution 0·01 M sodium-phosphate buffer. Penicillin-V (phenoxymethyl-penicillinic acid purchased from SIGMA Chemical, St Louis, USA) and urea (from Fluka BioChemika, Buchs, Switzerland) solutions were always prepared fresh in the above buffers before use. The enzyme used were; penicillinase [EC 3.5.2.6] from *Bacillus cereus* (Sigma Chemical) with a reported specific activity of 2300 units $mg^{-1}$ protein, where a unit is defined as the enzyme quantity that hydrolyses 1 μmole of benzylpenicillin to benzylpenicilloic acid per min at pH 7·0 at 25 °C and urease [EC 3.5.1.5] from *Jack Beans* (Sigma Chemical) with a reported specific activity of 782 units $g^{-1}$ solid, where a unit liberate 1 μmole of $NH_3$ from urea per min at pH 7·0 at 25 °C aqueous solution of glutaraldehyde (Sigma Chemical) was used as the cross-linking agent.

### *Procedure*

#### *Enzyme electrode preparation*

This was similar to that for the enzyme electrode described previously [15]. However, to immobilize penicillinase, the combined pH electrode was immersed in a penicillinase solution containing 4 mg $ml^{-1}$ of the enzyme. The concentration of the urease solution for electrode immersion solution was 16 mg $ml^{-1}$. The diaphragm of the reference electrodes was covered by plastic clips to prevent a contact with the enzyme and glutaraldehyde solution during the immobilization period.

#### *Flow-injection system*

The manifold is shown in figure 2. The base-line was obtained by pumping a buffer solution through the detection cell (pH meter in millivolt mode). The sample flow rate was always 0·78 ml $min^{-1}$. Penicillin standard solutions of 0·1 mM to 50 mM were prepared in the fermentation broth, urea standard solutions in the buffer. The diluted samples were injected into the carrier by means of the injection valve which is placed close to the detection cells in order to minimize the delay between injection and detection. The dead volume chosen was 2000 μl. The cells are thermostatted (25 °C). For measurements, the potential difference between the peak height and the base-line was automatically recorded by the computer and the recorder. The potential of the electrode always returned to its base-line when a fresh buffer solution came into contact with the electrode.

#### *Sampling of urea in human serum*

Serum samples, provided by the Laboratoire Medicale Fauriel (Saint-Etienne), were further centrifuged at 3000 rpm for 10 min. The pH-values of the samples were 7·75 ± 0·5.

#### *Sampling of penicillin in fermentation broth*

Penicillin in fermentation broth were measured after sample withdrawal from the bioreactor. The samples were filtered by an *in situ* membrane module with a 0·2 μm nominal pore diameter. The system is described elsewhere [14].

### Results and discussion

Measuring pencillin in fermentation broth and urea in human serum samples is important; penicillin fermentations typically run for 10 days and a high frequency of analysis is therefore unnecessary. However, this determination is done within a large concentation range and in a complex media. During the fermentation process there is a significant change in the medium composition, whereas the pH value of the broth remains approximately constant. The concentration range of urea, however, is smaller in human serum samples and a higher sample throughput is needed for routine clinical analysis. To fulfil these requirements, injection volume, flow rate and buffer strengths were adjusted to have good reproducibility, as well as acceptable sensitivity and response time.

### *Determination of the penicillin-V in fermentation broth*

#### *Linearity, sensitivity and reproducibility*

The electrode response to penicillin-V at four different injection volumes is shown in figure 3(*a*). Sensitivity is improved using a large injection volume. However, higher injection volumes produce a decrease in the linear concentration range and a lower injection rate. A good compromise between adequate sensitivity and linear range of response was obtained using a sample volume of 0·1 ml, which ensures a linear range between 0 and 100 mM. Consequently, the measurements during the whole fermentation time were done without changing any operating parameter.

Theoretically, the response to pencillin should be proportional to the logarithm of its concentration because the hydrogen ions ($H^+$) produced from the enzymic reaction are detected by a pH electrode, the response of which is related to the Nermst equation. In the absence of any enzymic catalysis, the recorded response for a batch system reflects the pH-change between the initial signal $E_0$ and the stationary response $E_{stat}$:

$$\Delta E = E_{stat} - E_0 = k_1 \log \Delta[H^+] \qquad (1)$$

where $[H^+]$ is the proton concentration change and $k_1$ the batch pH-operation constant factor for a given temperature respectively.

Logarithmic responses have been reported for batch measurements of penicillin by different authors [10,16,17]. In all cases the penicillinase enzyme was immobilized. If the enzymatic production of $H^+$ is proportionate to the substrate concentration (first order kinetics),

$$\Delta[H^+] = k_2[S] \qquad (2)$$

where $k_2$ is a constant factor for a given temperature, the response of the electrode is proportional to the logarithm of the substrate (penicillin) concentration:

$$\Delta E = k_3[S] \qquad (3)$$

where $k_3$ is the enzymic batch-operation constant factor.

However, using flow systems for potentiometric detection of penicillin, a linearity of the response to penicillin concentration has been observed [11,13]. This can be due to the fact that the batch steady state response is not reached with the FIA-system and so the Nernst equation is no longer valid. Figures 3(*b*) and 3(*c*) compare the FIA results with batch measurements obtained with a penicil-

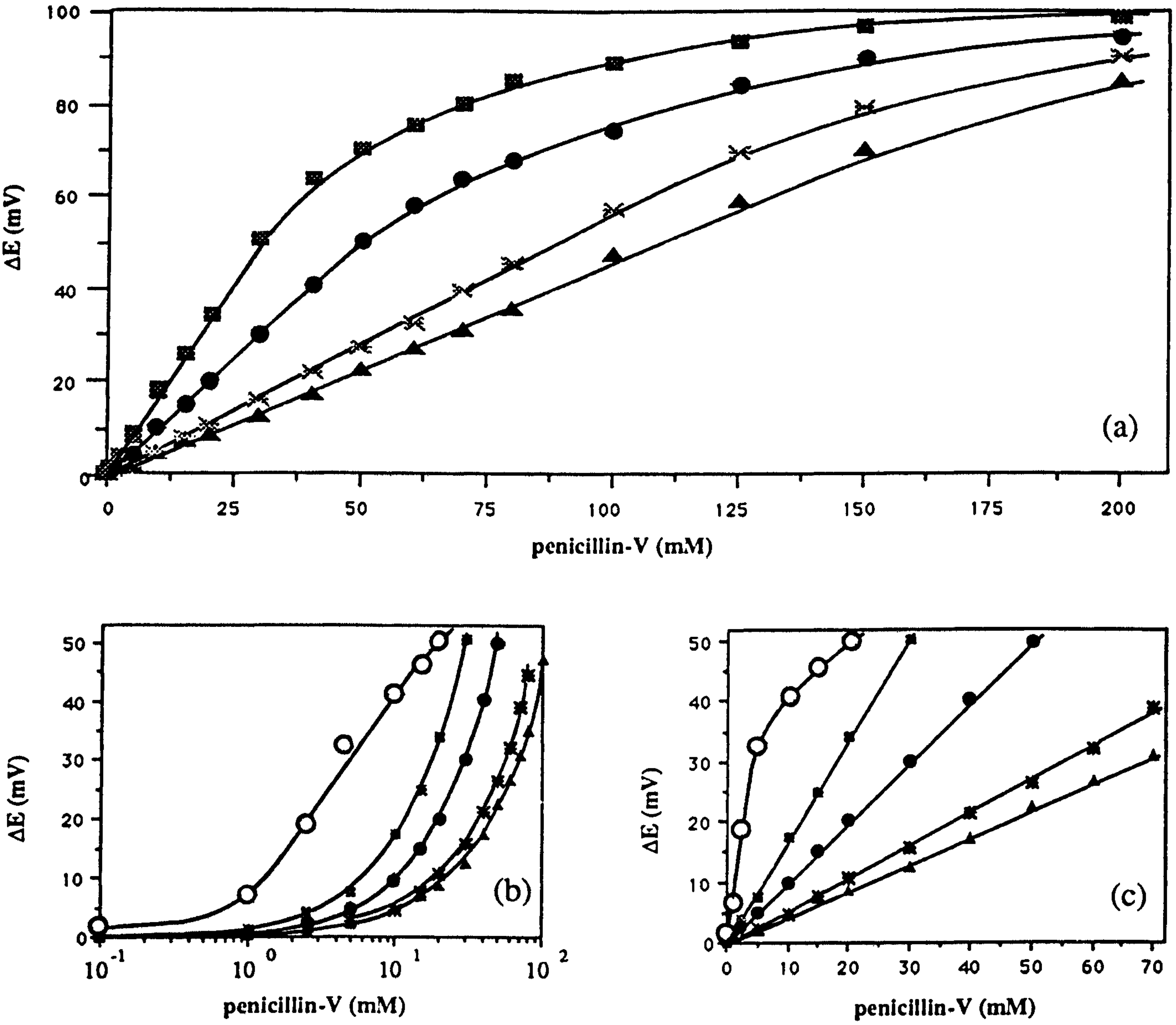

*Figure 3. (a) Calibration curves for penicillin-V obtained in fermentation broths; phosphate buffer used: 0·01 M/0·1 M NaCl (pH 6·5) at 25°C; flow rate: 1·1 ml min$^{-1}$; injection volumes: 50 µl (▲); 100 µl (★); 250 µl (●) and 500 µl (■); (b) Comparison of the FIA results with batch results (○) [10], tracing the penicillin-V concentration with a logarithmic axis. (c) Comparison of the FIA results with batch results (○) [10], tracing the penicillin-V concentration with a lineary axis.*

linase electrode [10]. Figure 3(*b*) shows the semi-logarithmic plotting as a function of the logarithm of the penicillin concentrations, whereas in figure 3(*c*) this is done as a linear function of the penicillin concentration. Figure 3(*b*) shows a typical Nernst response for batch measurement with a linear range from 1·5 to 15 mM. Figure 3(*c*) shows the direct linear dependence of the FIA peak heights to the penicillin concentrations. In this range the peak response for the FIA-system is given by

$$\Delta E = E_{\text{peak height}} - E_{\text{base line}} = k_4\,[\text{S}] \qquad (4)$$

where $k_4$ is the FIA-operation constant factor.

Reproducibility of the peaks obtained for penicillin fementation broth solutions was determined as shown in figure 4. The relative standard deviation (r.s.d.) of the measurements was 0·94%.

*Application during fed-batch penicillin-V fermentation*

Results of the analysis of penicillin-V fermentation broth samples were compared with results from an HPLC method [14]. The operating conditions chosen for FIA allow measurements without any change in parameters during the whole fermentation process. The pH of the broth is regulated to about 6·5 ± 0·2. Figure 5 shows the penicillin analysis for the fermentation FBO-13. The electrode response to penicillin-V is shown for two different injection volumes. The results demonstrate good agreement in biosensor responses over a wide range of concentrations with the HPLC measurements. The change of the composition of the fermentation medium with time affects the sensitivity of the biosensor. At the end of the fermentation time, the agreement between both detection methods is better for the smaller volume

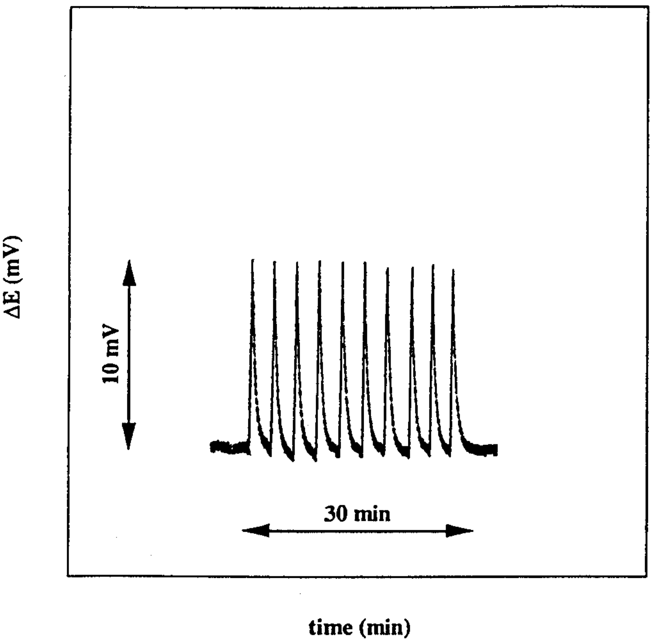

*Figure 4. Results for the reproducibility test for the penicillin electrode; concentration of penicillin-V in fermentation broths: 15 mM; phosphate buffer used: 0·01 M/0·1 M NaCl (pH 6·5) at 25°C; injection volume: 100 µl; flow rate: 4·0 ml min$^{-1}$.*

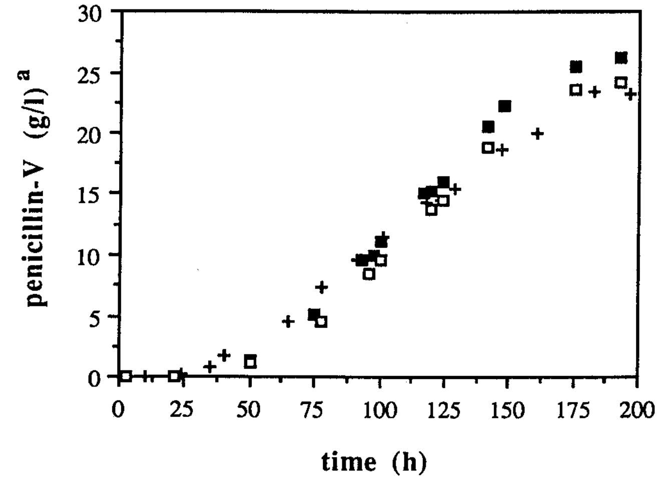

*Figure 5. Comparison of two different detection methods for the determination of penicillin-V in fermentation broths during the fermentation process: enzyme electrode incorporated in the FIA system and HPLC (+); injection volumes for the FIA system: 50 µl (□) and 100 µl (■); phosphate buffer used: 0·01 M/0·1 M NaCl (pH 6·5) at 25°C; flow rate: 1·1 ml min$^{-1}$; [a]1 M penicillin-V = 388 g.*

injected (50 µl) in the FIA system. This could be due to reduced interference with the broth elements by the biosensor using a higher dilution rate in the detection cell.

### *Automatic determination of urea*

#### *Linearity, sensitivity and reproducibility*

The electrode response to urea in the stirred-flow detection cell using the automated FIA system is shown in figure 6. Typical response peaks were recorded using a

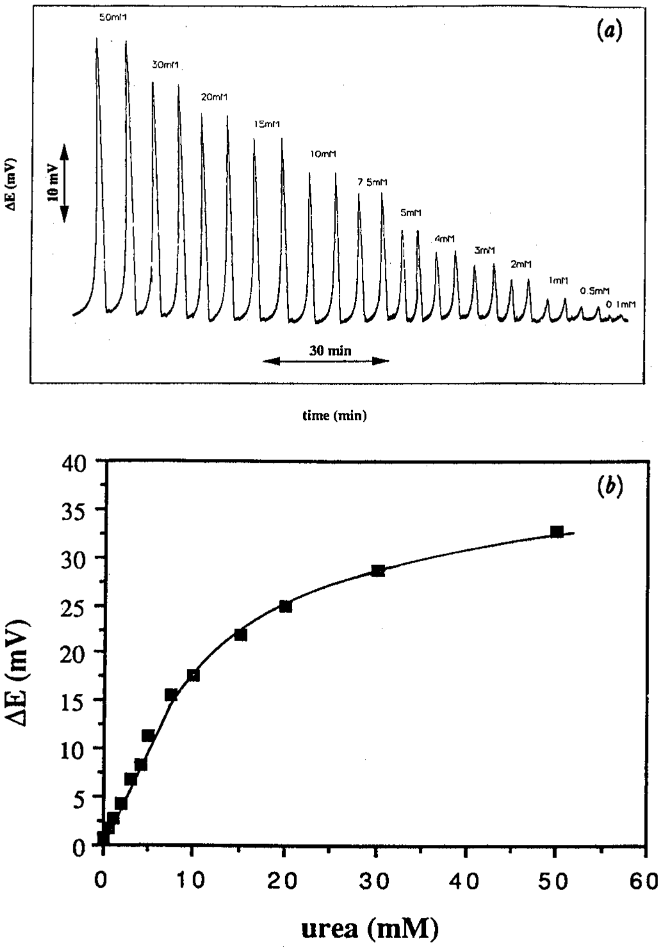

*Figure 6. (a) Calibration peaks for urea obtained in phosphate buffer 0·005 M/0·1 M NaCl (pH 7·75) at 25°C; injection volume: 100 µl; flow rate: 4·0 ml min$^{-1}$; (b) Calibration curve for urea obtained in phosphate buffer 0·005 M/0·1 NaCl (pH 7·75) at 25°C; injection volume: 100 µl; flow rate: 4·0 ml min$^{-1}$.*

flow rate of 4 ml min$^{-1}$ (figure 6(*a*)). From this measurement, the potential change $\Delta E$ is plotted against the urea concentration (figure 6(*b*)). Sensitivity is sufficient using a buffer at lower concentration, in comparison with the penicillin measurements. However, the linear range from 0 to about 10 mM is not large, but the normal urea concentrations in blood range from 2 to 8 mM [9].

Reproducibility of the peaks obtained for urea samples are shown in figure 7. The r.s.d. of the measurements was 1·94%.

#### *Measurement of urea in human serum samples*

The determination of the concentration of urea in serum samples in a clinical laboratory by a spectrophotometric method [18] and direct injection into the carrier stream of an FIA system are compared in figure 8. A good agreement is obtained between these two methods in the normal amounts of urea in blood.

#### *Stability of the biosensors*

The long-term stability of the two electrodes was examined at room temperature as shown in figure 9. After construction, two pencillinase and two urease electrodes

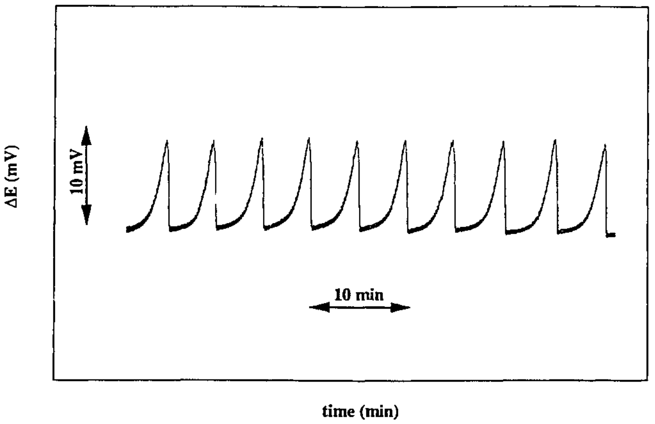

*Figure 7. Results for the reproducibility test for the urea electrode; concentration of urea in the buffer: 5 mM; phosphate buffer 0·005 M/0·1 M NaCl (pH 7·75) at 25°C; injection volume: 100 μl; flow rate: 4.0 ml min$^{-1}$.*

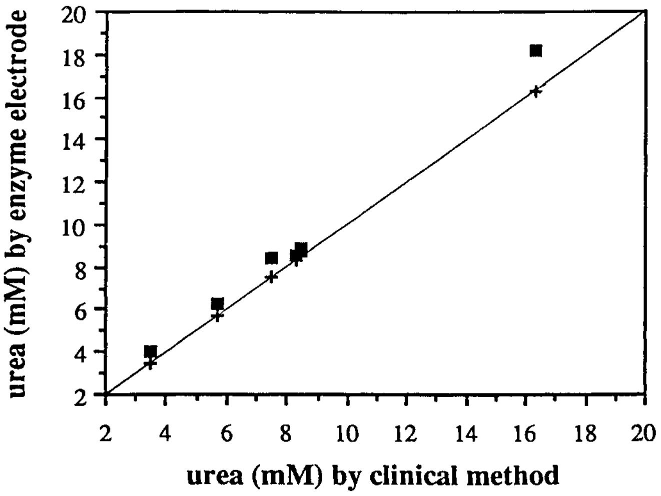

*Figure 8. Comparison of two different detection methods for the determination on urea in human serum samples: enzyme electrode incorporated in the FIA system (■) and spectrophotometric (+); phosphate buffer used: 0·005 M/0·1 M NaCl (pH 7·75) at 25°C; injection volume: 100 μl; flow rate: 4·0 ml min$^{-1}$.*

were used in the FIA system for about 8 hours per day. Between measurements, the sensors were stored in the detection cells at room temperature. The penicillinase electrode was stable over the test period of 33 days. However, the stability of the urease electrode due to the urease enzyme [15], is not as good.

### Conclusion

Using a new immobilization technique, it is possible to obtain a fast responding enzyme sensor using an ordinary pH glass electrode. This type of sensor can provide substrate concentrations after only a few seconds [15]. Automation and applications of the biosensor-FIA system have been described. Enzyme electrodes were directly incorporated in a home-made stirred flow

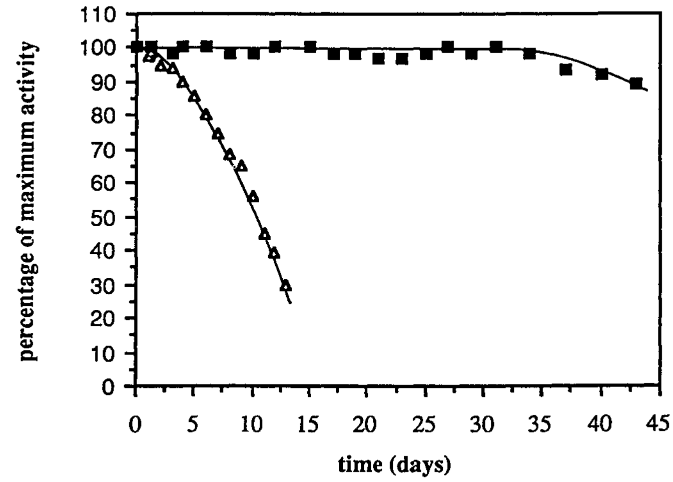

*Figure 9. Long-term stability of the penicillinase electrode (■) when stored in phosphate buffer (0·01 M/0·1 M NaCl, pH 6.5) and of the urease electrode (△) when stored in phosphate buffer (0·005 M/0·1 M NaCl, pH 7·75); substrate concentration: 5 mM; temperature: 25°C.*

detection cell. Various parameters influencing the electrode response were studied, and the performance of the penicillin sensor and the urea sensor were compared. The various conditions required for penicillin determination during the fermentation process and urea in human serum samples are reduced or completely eliminated by the sample dilution, which takes place in the detection cell. The optimal operating conditions of the FIA system have also been established.

The FIA system described here is simple, reliable and inexpensive and well-suited to the determination of urea in human serum samples and penicillin in fermentation broth samples.

### Acknowledgements

The authors wish to thank Dr J. Nielsen, Professor J. Villadsen and their research group at the Center for Bioprocess Research, Technical University of Denmark for both supplying the penicillin fermentation broth samples and for their support offered in this project. The authors also wish to thank Dr Meley (Laboratoire d'Analyses Médicales, Saint-Etienne) for supplying the human serum samples, and Mr G. Grawley.

### References

1. Nielsen, J., Nikolajsen, K. and Villadsen, J., *Biotechnology and Bioengineering*, **33** (1989), 1127.
2. Meier, H. and Tran-Minh, C., *Analytica Chimica Acta*, **264** (1992), 13.
3. Solich, P., Polasek, M. and Karlicek, R., *Analytica Chimica Acta*, **218** (1989), 151.
4. Kiba, N., Tagami, H. and Furusawa, M., *Analytica Chimica Acta*, **239** (1990), 307.
5. Van Opstal, M. A. J., Wolters, R., Blauw, J. S., Van Krimpen, P. C., Van Bennekom, W. P. and Bult, A., *Journal of Pharmaceutical and Biomedical Analysis*, **8** (1990), 49.

6. Kumaran, S. and Tran-Minh, C., *Analytical Biochemistry,* **200** (1992), 187.

7. Ruzicka, J. and Hansen, E. H., *Flow Injection Analysis* (Interscience-Wiley, New York, 1981).

8. Ruzicka, J., Hansen, E. H., Ghose, A. K. and Mottola, H. A., *Analytical Chemistry,* **51** (1979), 199.

9. Narainesingh, D., Mungal, R. and Ngo, T. T., *Analytical Biochemistry,* **188** (1990), 325.

10. Meier, H., Kumaran, S., Danna, A.-M. and Tran-Minh, C., *Analytica Chimica Acta,* **249** (1991), 405.

11. Rushling, J. F., Luttrell, G. H., Cullen, L. F. and Papariello, G. J., *Analytical Chemistry,* **48** (1976), 1211.

12. Gnanasekaran, R. and Mottola, H. A., *Analytical Chemistry,* **57** (1985), 1005.

13. Olsson, B., *Analytica Chimica Acta,* **209** (1988), 123.

14. Christensen, L. H., Nielsen, J. and Villadsen, J., *Analytica Chimica Acta,* **249** (1991), 123.

15. Kumaran, S., Meier, H., Danna A.-M. and Tran-Minh, C., *Analytical Chemistry,* **63** (1991), 1914.

16. Papariello, G. J., Mukherji, A. K. and Shearer, C. M., *Analytical Chemistry,* **45** (1973), 790.

17. Cullen, L. F., Rushling, J. F., Schleifer, A. and Papariello, G. J., *Analytical Chemistry,* **46** (1974), 1955.

18. Neumann, U. and Ziegenhorn, J., XVI. Nordiska kongressen for klinisk kemi och klinisk fysiologi (1977), Oulu, Finland.

